# Clinical Equipoise and Unimproved Outcomes following Posttransplant Cutaneous Squamous Cell Carcinoma

**DOI:** 10.1016/j.ekir.2026.106522

**Published:** 2026-04-01

**Authors:** Tom H.J. Crisp, Emilia Peleva, Rachel Abbott, Rabiah Ahmed, Charles Archer, Adarsh Babu, Philippa Bailey, Nitin Bhandary, Jeva Cernova, Amrit Darvay, Jonathan Gamble, Maria Gauci, Sian Griffin, Emily Karn, Rubeta Matin, James Moriarty, Nina Muirhead, Khizr Nawab, Alex Owen, Emily Ruiz, Farida Shah, Adnan Sharif, Brenda Solomon, Raj Thuraisingham, Harry Wakefield, Elizabeth Wallin, Buddhika Wijayawickrama, Catherine A. Harwood, Matthew J. Bottomley

**Affiliations:** 1 Oxford Kidney and Transplant Unit, Churchill Hospital, Oxford University Hospitals NHS Foundation Trust, Oxford, UK; 2 Center for Cancer Evolution, Barts Cancer Institute, Queen Mary University of London, London, UK; 3 Department of Dermatology, Royal London Hospital, Barts Health NHS Trust, London, UK; 4 Department of Dermatology, Cardiff and Vale University Health Board, Cardiff, UK; 5 Department of Nephrology, University Hospitals Birmingham NHS Foundation Trust, Birmingham, UK; 6 Department of Renal Medicine, Royal Berkshire NHS Foundation Trust, Reading, UK; 7 Department of Nephrology, Gloucestershire Hospitals NHS Foundation Trust, Gloucester, UK; 8 Department of Nephrology, North Bristol NHS Trust, Bristol, UK; 9 Mohs and Dermatologic Surgery Center, Brigham and Women's Hospital, Boston, Massachusetts, USA; 10 Department of Dermatology, Oxford University Hospitals NHS Foundation Trust, Oxford, UK; 11 Department of Dermatology, Buckinghamshire Healthcare NHS Trust, Aylesbury, UK; 12 Department of Nephrology, Royal London Hospital, Barts Health NHS Trust, London, UK; 13 Center for Cell Biology and Cutaneous Research, Faculty of Medicine and Dentistry, Blizard Institute, Queen Mary University of London, London, UK; 14 CAMS Oxford Institute, Chinese Academy of Medical Sciences & Peking Union Medical College, University of Oxford, Oxford, UK; 15 Department of Nephrology, Cardiff and Vale University Health Board, Cardiff, UK; 16 Department of Dermatology, University Hospitals Birmingham NHS Foundation Trust, Birmingham, UK; 17 Department of Dermatology, Royal Berkshire NHS Foundation Trust, Reading, UK; 18 Department of Dermatology, Gloucestershire Hospitals NHS Foundation Trust, Gloucester, UK; 19 Department of Dermatology, North Bristol NHS Trust, Bristol, UK

**Keywords:** cancer, clinical study, immunosuppression, skin cancer, transplantation

## Abstract

**Introduction:**

Cutaneous squamous cell carcinoma (CSCC) is the most common malignancy following solid organ transplantation. Historical data suggest that 25% of kidney transplant recipients (KTRs) develop additional CSCC within a year of their first lesion, with significantly increased risks of metastasis and mortality. However, contemporary outcomes and optimal secondary prevention strategies remain unclear.

**Methods:**

We conducted a multicenter retrospective cohort study across 8 UK transplant centers, identifying 136 KTRs diagnosed with first-ever CSCC between 2016 and 2020.

**Results:**

Over a median follow-up of 39 months, 48.5% developed further CSCC, and 23.3% died, most commonly from malignancy. Poor outcomes were confirmed in another, international cohort. Management varied within and between UK centers and 28.7% underwent immunosuppression reduction, though specific approaches were inconsistent. Distinct clinical and histopathological features were associated with recurrence risk and poorer outcomes, including multiple index lesions, high-risk histopathology, and current smoking.

**Conclusion:**

These findings confirm that contemporary post-CSCC outcomes remain unchanged from historic outcomes, despite advances in transplant care. The variability in management after first CSCC highlights the need for prospective studies to define effective interventions for secondary prevention. Importantly, we identify important clinical features present at first CSCC that may guide the targeting of such interventions to the highest risk patients.

## Graphical abstract

**Figure:**
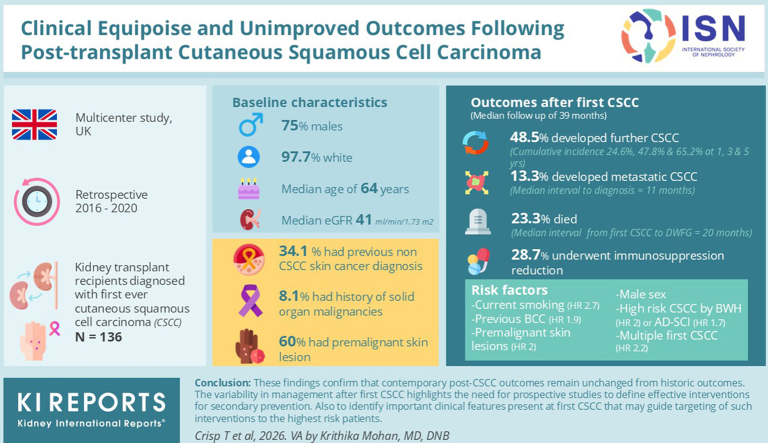

Despite improvements in short-term outcomes, long-term survival after kidney transplantation remains suboptimal, with half of recipients dying with a functioning graft; often owing to malignancy.^1^, ^2^, ^3^ Skin cancer, 80% of which is CSCC, is the most common posttransplant malignancy, affecting up to half of all solid organ transplant recipients (SOTRs).^4^^,^^5^ Risk factors include lighter skin tone and increasing age, cumulative immunosuppression duration, and ultraviolet radiation exposure.^6^

Historical data suggest that 25% of SOTRs with a first (primary) CSCC develop another within a year, increasing to > 60% at 5 years.^7^^,^^8^ Multiple posttransplant CSCC are associated with increased risk of metastasis and other malignancies.^5^^,^^8^, ^9^, ^10^ Changes in transplant demographics and immunosuppressive regimens over the past 2 decades may influence CSCC outcomes; however, their impact remains unclear.^11^, ^12^, ^13^, ^14^, ^15^, ^16^

Uncertainty exists regarding optimal strategies for secondary prevention of further disease, largely because of a paucity of prospective data and limitations in existing studies. Most cohort studies in our subject population are single-center, affected by era bias, and lack granularity.^17^ Potential interventions either face limited real-world applicability despite promising risk reduction, such as difficulty using mammalian target of rapamycin inhibitors (mTORi) because of patient intolerance,^4^^,^^18^, ^19^, ^20^ or are underpinned by conflicting evidence, as exhibited by studies investigating nicotinamide as chemoprevention.^21^^,^^22^ Moreover, the absence of robust risk stratification tools hampers the ability to tailor secondary prevention approaches effectively.^17^ Contemporary data on treatment strategies following first CSCC in SOTRs remain sparse, further contributing to uncertainty in decision-making.

We hypothesize that this evidence gap has led to wide variation in secondary prevention practices. The Contemporary Outcomes After Skin cancer in Transplant (COAST) study evaluates current management and outcomes following first CSCC in KTRs.

## Methods

COAST is a multicenter, retrospective cohort study identifying patients from 8 renal centers, including 5 transplanting centers, in southern England and Wales. Centers are anonymized for reporting. All consecutive adults with a functioning kidney (or kidney and pancreas) transplant at time of their first-ever CSCC, diagnosed between January 1, 2016 and December 31, 2020, were included. Patients (cases) were sourced by cross-referencing histopathological databases or skin cancer multidisciplinary team databases with transplant databases. We excluded patients with previous diagnosis of CSCC, or history of other organ or stem-cell transplant. In the UK, CSCCs are discussed at multidisciplinary team meetings as part of standard care. After diagnosis of CSCC, KTRs remain under regular dermatology surveillance, with follow-up intervals determined by individual risk factors.^8^ Follow-up began on the date of the first CSCC and finished at the last clinic appointment (transplant or dermatology) if lost to follow-up, death, or December 31, 2022 (whichever occurred first). Patients were additionally censored in the event of graft loss, defined as initiation of dialysis.

Clinicopathological data were collected from local electronic patient records using a standardized data collection proforma (Supplementary Table S1). Data were cross-checked by a nephrologist and dermatologist in the coordinating center before anonymization. CSCCs were retrospectively classified using the Brigham and Women’s Hospital (BWH) staging and the eighth edition American Joint Committee on Cancer (AJCC8)/International Union Against Cancer (UICC8) staging system.^23^^,^^24^ Lesions were additionally classified according to the Actinic Damage and Skin Cancer Index^25^ and the British Association of Dermatologists’ CSCC guidelines.^26^ Multiple classifications were included because of limited data on their performance in immunosuppressed cohorts and uncertainty regarding the criteria for optimal risk prediction. Where multiple index lesions were excised, the highest risk lesion (by BWH classification) was used for survival analyses. Comprehensive data on ultraviolet exposure was not collected as it is not routinely available.

Early management was defined as any documented change in therapy within 6 months of CSCC diagnosis. Immunosuppression intensity was characterized as decreased (“immunosuppression reduction,” ISR) if dose reduction or cessation of an agent was undertaken. A switch of agent was recorded but considered as unchanged immunosuppression intensity. Initiation of topical treatments for premalignant actinic keratoses with potential chemopreventative activity (5-fluorouracil, imiquimod), destructive therapies (cryotherapy/curettage/cautery), and systemic chemoprevention (nicotinamide and acitretin) within this timeframe were also recorded.

An international cohort was assembled to validate the COAST study findings regarding outcomes following a first CSCC. All adult SOTRs, with a first-ever CSCC diagnosed at the Brigham and Women’s Hospital, USA, between January 1, 2012 and December 31, 2020 were included. The follow-up period was calculated as the date of the first CSCC histology report to the last skin cancer–related clinic, death, or April 9, 2025 (whichever occurred first).

### Statistical Analysis

Descriptive statistics were used to summarize clinical and histopathological characteristics. Continuous variables were reported using median and interquartile range (IQR) for nonparametric data and mean and SD for parametric data. Baseline characteristics and management strategies across the 8 study centers were compared using Pearson’s chi-square test with a Monte Carlo simulation. Association between baseline characteristics and early ISR was assessed using the Mann–Whitney U test for continuous variables and Fisher exact test for categorical variables. Statistically significant factors were evaluated further using multivariate logistic regression modelling.

Survival and time-to-event data were analyzed using the “survival” package in R (R Foundation for Statistical Computing, Vienna, Austria) and visualized using Kaplan-Meier univariate modelling. Kaplan-Meier curves were used to estimate the cumulative incidences of further CSCC and poor outcomes. Influence of clinicopathological variables on patient outcomes was assessed using Cox proportional hazards models, constructed based on prespecified clinical hypotheses and findings from exploratory univariate analyses. Study outcomes were “further CSCC” development, and a composite “all poor outcomes,” including graft loss, further CSCC, nonkeratinocyte, solid-organ malignancy (SOM), metastatic CSCC, or death with a functioning graft (DWFG). Further CSCC was defined as a new primary CSCC and recurrences of previous CSCC were excluded. In the case of further CSCC, multivariate modelling was conducted with competing risk analysis, applying the Fine-Gray method for subdistribution proportional hazards modelling implemented using the “cmprsk” R package. Death and graft loss were treated as competing risks.^27^ Results are reported as hazard ratios (HRs) with 95% confidence intervals. As a sensitivity analysis, survival models for the composite poor outcome were repeated after excluding further CSCC as an event (“poor outcomes without CSCC”).

Statistical significance was set at 0.05 (2-sided). Statistical analysis was performed using R version 4.3.2 via RStudio, using the most recent versions of the appropriate packages.^28^ All plots were created using the “ggplot2” package.

This report was written in accordance with the Strengthening the Reporting of Observational Studies in Epidemiology statement (Supplementary Table S2).^29^

### Ethical Approval

The COAST study was conducted in accordance with the Declaration of Helsinki and was approved by the NHS Human Research Authority (Ethics reference:22/HRA/3782). Participant consent was waived because the study used routinely collected clinical information only.

## Results

**Figure 1:**
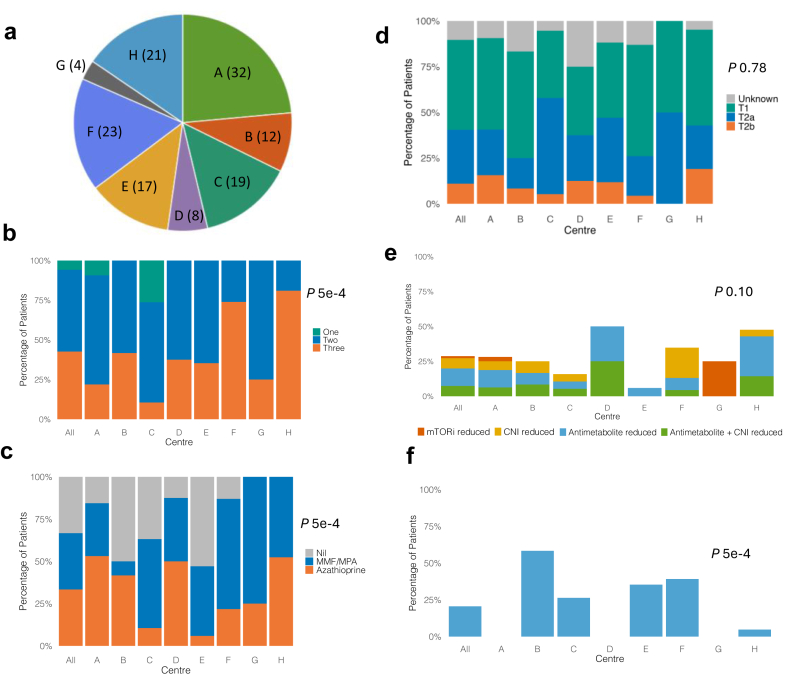
Baseline characteristics and early management after a first cutaneous squamous cell carcinoma. (a) Pie chart shows number of patients per center, with centers labelled A to H; 136 patients were included in total. Stacked bar plots show baseline characteristics at time of first cutaneous squamous cell carcinoma, including (b) number of immunosuppressive agents, (c) antiproliferative agents used, and (d) Brigham and Women’s Hospital classification stage of first cutaneous squamous cell carcinoma. Results are shown for the entire cohort (all) and for each center (A–H). (e) Stacked bar plot shows immunosuppression reduction approach for the entire cohort (“all”) and by center. Overall, immunosuppression was reduced in 28.7% of patients (range: 5.9%–50.0% by center). (f) Bar plot shows initiation of topical therapy for the entire cohort (20.6%) and by center (range: 0%–58.3%). 5-fluorouracil cream was started in all but one case, in whom imiquimod cream was used. CNI, calcineurin inhibitor; MMF, mycophenolate mofetil; MPA, mycophenolic acid; MTORi, mammalian target of rapamycin inhibitors.

Data were initially provided for 153 patients; 17 were excluded as ineligible after review (Supplementary Table S3), leaving 136 eligible patients for downstream analysis (Figure 1a).

### Baseline Characteristics

In Table 1, we summarize cohort features at time of first CSCC. Notably, most patients were male (75.0%), White (97.7%) and had a median (IQR) age of 64 (57–72) years. The median estimated glomerular filtration rate was 41 (27–55) ml/min per 1.73 m^2^.

**Table 1:** Baseline characteristics of 136 patients diagnosed with first cutaneous squamous cell carcinoma (CSCC)

Baseline Characteristics	All patients (*N* = 136)
Age (yrs)	64 (57–72)
Sex	
Male	102 (75.0)
Female	34 (25.0)
Ethnicity	
White	128/131 (97.7)
Black	2/131 (1.5)
Asian	1/131 (0.7)
Previous solid-organ malignancy^a^	11/135 (8.1)
Previous non-cSCC skin cancer^b^	46/135 (34.1)
Previous actinic keratoses^c^	67 (49.3)
Previous Bowen’s/keratoacanthoma^d^	43/135 (31.9)
Smoking history	
Current	11/63 (17.5)
Exsmoker	15/63 (23.8)
Never smoker	37/63 (58.7)
Total number of transplants	
1	107 (78.7)
2	23 (16.9)
3	6 (4.4)
eGFR	41 (27–55)
Previous rejection	21/134 (15.7)
Evidence of DSA before CSCC	11/134 (8.2)
Cumulative lifetime duration of IS (mos)	136 (68-204)
Induction therapies	
Basiliximab/equivalent	68/118 (57.6)
Alemtuzumab	21/118 (17.8)
ATG/thymoglobulin	6/118 (5.1)
Other	4/118 (3.4)
None	19/118 (16.1)
Number of IS agents at diagnosis	
1	8 (5.9)
2	69 (50.7)
3	59 (43.4)
Calcineurin/mTOR inhibitor	
Tacrolimus	104 (76.5)
Ciclosporin	18 (13.2)
Sirolimus	4 (2.9)
Nil	10 (7.4)
Antiproliferative	
MMF/MPA	59 (43.4)
Azathioprine	46 (33.8)
Nil	31 (22.8)
Corticosteroid	92 (67.6)
Number of CSCC removed at first episode	
Single	127 (93.4)
Multiple	9 (6.6)

ATG, antithymocyte globulin; CSCC, cutaneous squamous cell carcinoma; DSA, donor-specific antibody; eGFR, estimated glomerular filtration rate; IS, immunosuppression; MMF, mycophenolate mofetil; MPA, mycophenolic acid; mTOR, mammalian target of rapamycin.

a 10/11 cancers were in remission at study baseline. (Supplementary Table S8)

b Previous basal cell carcinoma (*n* = 44) and/or melanoma (*n* = 3).

c Includes both clinical and histopathological diagnoses.

d Previous Bowen’s disease (CSCC-*in situ*, histologically diagnosed in 37 patients and clinically diagnosed by a dermatologist in 4 patients) or keratoacanthoma (histologically diagnosed in 3 patients). Values are expressed as number (percentage) or median (interquartile range), as appropriate. Where data are missing, results have been expressed as a fraction.

One-third of the cohort (34.1%) had a previous non-CSCC skin cancer diagnosis (predominantly, basal cell carcinoma, BCC) whereas 11 patients (8.1%) had a history of SOM. Over half (60.0%) had a previously documented clinical or histopathological diagnosis of a premalignant skin lesion including actinic keratosis, Bowen’s disease (CSCC-*in situ*) or keratoacanthoma. Comparing baseline characteristics across centers, there were significant differences (*P* < 0.05, chi-square test) in the proportions of patients with previous rejection episodes, immunosuppression regimens (induction therapy, antiproliferative, steroid use, and number of agents), year of first CSCC (2016–2020), and multiple CSCC. Biological sex, smoking status, and history of BCC were not significantly different between centers (Supplementary Table S4).

### Immunosuppression at First CSCC

The median cumulative duration of immunosuppression at first CSCC was > 11 years (136 months, IQR: 68–204 months). Over 80% of patients, where this information was available, had previously received induction therapy, with the majority receiving CD25-targetting monoclonal antibodies. Maintenance immunosuppression regimens varied significantly across centers (Figure 1b and c, Supplementary Table S4) in terms of number of agents, antiproliferative and corticosteroid use (*P:* 5 × 10^−4^, chi-square test). Patients were most commonly on 2 or 3 immunosuppressive agents at CSCC diagnosis. Most KTRs (89.7%) were taking a calcineurin inhibitor, most commonly, tacrolimus (76.5%). Those on tacrolimus had a median recent trough level of 6.2 (5−7.5) ng/ml and 22 patients had had a tacrolimus level > 8 ng/ml. The majority (77.2%) were taking an antiproliferative agent, most commonly a mycophenolic acid−based preparation (43.4%). Overall, only one-third of patients were receiving azathioprine. Two-thirds were receiving oral corticosteroids at first CSCC.

### Histopathological Characteristics of First CSCC

First CSCC histopathological characteristics are presented in detail in Table 2. Nine KTRs (6.6%) had multiple CSCC at their index presentation and 147 tumors in total were excised, most frequently located on the head-and-neck (63.3%) and upper limb (25.2%). Four-fifths of tumors had clear excision margins, increasing to 92.8% following reexcision. There was a discrepancy in risk classification depending on the system used: 14 tumors (10.4%) were high-risk by both BWH (defined as ≥ T2b) and AJCC8 (≥ pT3); 17 tumors (12.7%) were high-grade by AJCC8 only (of which 11 were T2a and 6 were T1 by BWH); 1 (0.8%) was high grade by BWH only (AJCC8 pT2). The proportion of patients with a first “high-grade” CSCC varied across centers (AJCC8 range: 5%–50% and BWH 0%–20%, Figure 1d), although this did not reach statistical significance (*P* = 0.23 and 0.75 for AJCC8 and BWH, respectively, chi-square test).

**Table 2:** Characteristics of first cutaneous squamous cell carcinoma (CSCC)

Characteristics	Number of tumors (*n* = 147)
Location	
Head and neck	93 (63.3)
Trunk	12 (8.2)
Upper limb	37 (25.2)
Lower limb	5 (3.4)
Diameter (mm)	12 (9–20)
Depth (mm)	3 (1.9–5)
Invasion beyond subcutaneous fat	8/128 (6.3)
Differentiation	
Well	40/145 (27.6)
Moderate	78/145 (53.8)
Poor	27/145 (18.6)
PNI	8/130 (6.2)
LVI	3/141 (2.1)
Clear margins after excision	117/146 (80.1)
Clear margins after re-excision	128/139 (92.8)
BWH	
T1	77/133 (57.9)
T2a	41/133 (30.8)
T2b	15/133 (11.3)
AJCC8/UICC8	
pT1	91/134 (67.9)
pT2	11/134 (8.2)
pT3	32/134 (23.9)
AD-SCI	
4	85/134 (63.4)
5	16/134 (11.9)
6	33/134 (24.6)
BAD	
High risk	95 (70.4)
Very high risk	40 (29.6)

AD-SCI, Actinic Damage and Skin Cancer Index; AJCC8/UICC8, the 8th edition of the American Joint Committee on Cancer/International Union Against Cancer staging systems; BAD, British Association of Dermatologists’ CSCC guidelines; BWH, Brigham and Women’s Hospital classification; LVI, lympho-vascular invasion; PNI, perineural invasion.

Values are expressed as number (percentage) or median (interquartile range), as appropriate.

Where data are missing, results have been expressed as a fraction.

### Early Management After CSCC

ISR was undertaken within 6 months of first CSCC in 39 patients (28.7%). The agents reduced included antiproliferatives (*n* = 17, 43.6%), calcineurin inhibitor (*n* = 10, 25.6%) and mTORi (*n* = 2, 5.1%). In addition, 10 patients (25.6%) had both antiproliferative and calcineurin inhibitor reduced. Only 1 patient was converted to an mTORi. CSCC was the most common reason cited for ISR (*n* = 21, 53.8%). Other cited reasons included undesirable trough levels (*n* = 13, 33.3%), recurrent infections (*n* = 4, 10.3%), and adverse effects (*n* = 3, 7.7%).

ISR frequency varied significantly among patients on different drug regimens: ISR was observed in 21 of 46 (45.7%) patients receiving azathioprine, 13 of 59 (22.0%) receiving mycophenolate-based regimens, 35 of 122 (28.9%) receiving calcineurin inhibitor, and 3 of 4 (75%) receiving mTORi (*P:* 0.01, Fisher test).

Factors independently associated with early ISR on multivariate analysis (Table 3) were diagnosis with a high-grade first CSCC (BWH > T2b, odds ratio: 4.6, *P*: 0.01) and receiving azathioprine (OR 3.33, P 0.003), whilst those with a previous BCC were less likely to undergo ISR (odds ratio: 0.17, *P*: 0.0005, Fisher test). There was no statistically significant difference in the proportions of patients who underwent early ISR across study centers (*P*: 0.10, chi-square test); however, the pattern of ISR varied within and between centers (Figure 1e). A center’s transplanting status did not significantly affect frequency or type of ISR (*P*: 0.36 and *P*: 0.06, Fisher test, respectively).

**Table 3:** Factors associated with early reduction in immunosuppression

	Odds ratio	*P*-value	LRM estimate	*P*-value
Male	0.56	0.19		
Age ≥ 65 yrs	0.84	0.71		
Current smoker	2.06	0.30		
Previous solid-organ malignancy	2.19	0.30		
Previous BCC	0.17	0.0005^a^	−1.70	0.006^a^
Previous transplants	1.72	0.25		
eGFR	W = 2242	0.09	−0.02	0.20
Previous rejection episode	0.53	0.31		
Evidence of DSA prior to first CSCC	0.23	0.18		
Perineural invasion	4.21	0.05		
Incomplete excision margins^a^	3.65	0.01^a^		
BWH	4.60	0.01^a^	1.40	0.03^a^
AJCC8/UICC8	2.28	0.07		
AD-SCI	2.11	0.09		
BAD	2.27	0.05		
Multiple first CSCC	1.26	0.72		
Azathioprine	3.33	0.003^a^	1.17	0.01^a^
Early further CSCC	1.88	0.32		
Study center	NA	0.08		
Year of first CSCC	NA	0.12		

AD-SCI, Actinic Damage and Skin Cancer Index, AJCC8/UICC8, the 8th Edition of the American Joint Committee on Cancer/International Union Against Cancer staging systems, BAD, British Association of Dermatologists’ CSCC guidelines; BCC, basal cell carcinoma; BWH, Brigham and Women’s Hospital classification; CSCC, cutaneous squamous cell carcinoma; eGFR, estimated glomerular filtration rate; DSA: donor-specific antibodies; LRM: multivariate logistic regression modelling.

a No longer significant (P: 0.21) when reexcision margins were taken into account. The association between clinicopathological factors and having early reduction in immunosuppression were evaluated using Fisher exact test, except eGFR at first CSCC which was assessed using the Mann-Whitney U test. Factors which were statistically significant were evaluated further using multivariate logistic regression modelling. Variables included baseline characteristics at the time of the first CSCC including: male sex, age ≥ 65 years (selected based on the median age of cohort), current smoker compared with never-smoker, previous solid-organ malignancy, previous BCC, previous transplants (prior to current transplant), eGFR (nearest measurement to time of first CSCC), previous rejection episode, evidence of DSA prior to first CSCC, perineural invasion (of the first CSCC), incomplete excision margins (before reexcision), BWH (grades ≥ T2b), AJCC8/UICC8 (grades ≥ pT3), AD-SCI (grades 5-6), BAD (‘Very high’ risk), multiple first CSCC, azathioprine (compared to mycophenolic-acid based therapies or no antiproliferative), early further CSCC (having a further CSCC within 6 mos), study center, and year of first CSCC (diagnosed 2016–2020).

Twety-eight patients (20.6%) had documented initiation of topical therapies within 6 months of first CSCC, of which all but 1 started 5-fluorouracil. The frequency of initiation varied significantly across centers (Figure 1f, *P*: 5 × 10^−4^, chi-square test). Six patients (4.4%) started systemic chemoprevention (nicotinamide, *n* = 4; acitretin, *n* = 2).

### Outcomes After First CSCC

**Figure 2:**
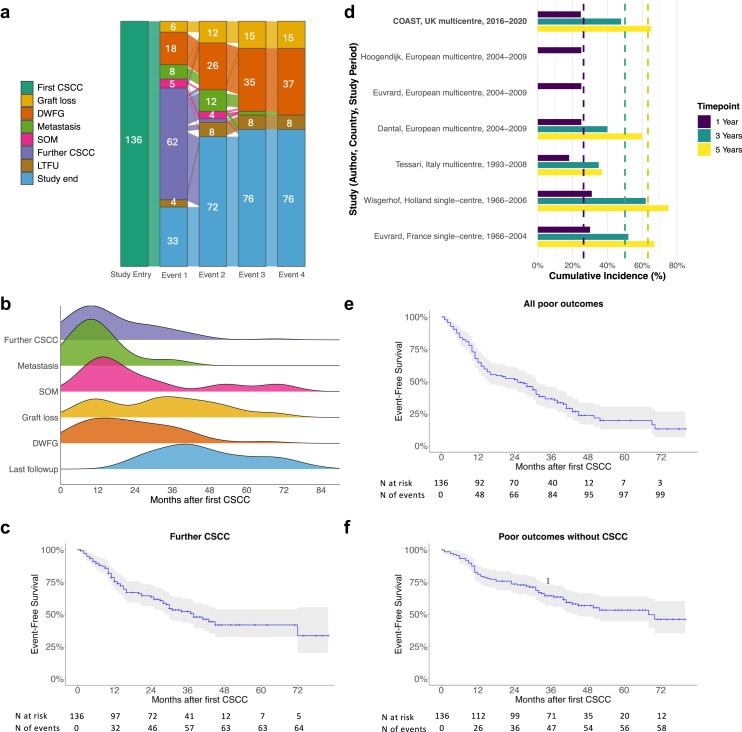
Outcomes after first CSCC in kidney transplant recipients. (a) Alluvial plot shows sequence of events after the first CSCC, including graft loss, DWFG, metastasis, SOM, further CSCC, or censoring due to LTFU or study end. (b) Stacked ridgeline plot shows time from diagnosis of first CSCC to development of poor outcomes and to last follow-up (last clinic appointment before loss to follow-up or study end). (c) Kaplan-Meier curve shows time to further CSCC. (d) Bar plot shows cumulative incidence of further CSCC at 1, 3, and 5 years after a first-ever CSCC in kidney transplant recipients in the present study (The Contemporary Outcomes After Skin cancer in Transplant study) and in historical published cohorts. The dashed lines show the weighted averages, based on the number of participants in each study. (e) Kaplan-Meier curve shows time to the composite “all poor outcomes.” (e) Kaplan-Meier curve shows time to “poor outcomes without CSCC.” CSCC, cutaneous squamous cell carcinoma; DWFG, death with a functioning graft; LTFU, loss to follow-up; SOM, solid organ malignancy.

Study outcomes are shown in Table 4 and summarized in Figure 2a and b. During a median (IQR) follow-up of 39 (26–52) months, 66 patients (48.5%) developed further CSCC, with tumor burden ranging from 1 to 13 (median 2) further lesions. The estimated cumulative incidence (95% confidence interval) of further CSCC was 24.6% (16.8%–31.6%), 47.8% (37.7%–56.3%), and 65.2% (45.9%–77.6%) at 1, 3, and 5 years, respectively (Figure 2c), with a median (IQR) interval between first and second CSCC of 13 (8–27) months. This cumulative incidence is comparable to historical studies (Figure 2d).^7^^,^^8^^,^^30^

**Table 4:** Outcomes during follow-up

Outcome	All patients (*N* = 136)
Follow-up (mos)	39 (26–52)
All poor outcomes^a^	99 (72.8)
Death	38 (23.3)
Time to death (mos)	20 (11–32)
Causes of death	
CSCC-related	11 (28.9)
Infection	6 (15.8)
CVD	6 (15.8)
Other malignancy	2 (5.3)
Decompensated liver disease	1 (2.6)
Other	2 (5.3)
Unknown	10 (26.3)
Graft loss	17 (12.5)
Time to graft loss (mos)	34 (18–45)
Biopsy-proven rejection	1/132 (0.8)
New DSA	3/110 (2.7)
Further CSCC	66 (48.5)
Median time to further CSCC (mos)	13 (8–27)
Metastatic CSCC	18/135 (13.3)
Time to metastatic CSCC (mos)	11 (7–12)
Solid-organ malignancy^b^	10 (7.4)
Time to solid-organ malignancy (mos)	16 (14–27)

CSCC, cutaneous squamous cell carcinoma; CVD, cardiovascular disease; DSA, donor-specific antibody.

a Composite outcome including graft loss, new CSCC, solid-organ malignancy, metastasis, or death with a functioning graft.

b Solid organ malignancies included gastro-intestinal malignancy (*n* = 3), cancer of transplanted kidney (*n* = 2), posttransplant lymphoproliferative disorder (*n* = 2), breast cancer (*n* = 1), tonsillar carcinoma (*n* = 1), pleomorphic dermal sarcoma (*n* = 1), and pseudomyxoma peritonei (*n* = 1). Values are expressed as number (percentage) or median (interquartile range), as appropriate.

Eighteen patients (13.3%) developed metastatic CSCC, with 15 cases confirmed on histopathology and 3 diagnosed on imaging. Eleven had metastases limited to the parotid gland and lymph nodes, whereas the remaining 7 had distant spread. One-third of CSCC metastases (*n* = 6) occurred in patients with a solitary prior CSCC (83.3% of these were high-risk CSCC). Median (IQR) interval to metastasis diagnosis was 11 (7–12) months, with 89% of patients developing metastases within 2 years of their first CSCC. Estimated CSCC-specific mortality after metastasis was 42.8 % and 75.5% at 1 and 3 years, respectively.

Thirty-eight patients (23.3%) died during follow-up, representing the most common cause of premature censoring. The median (IQR) time from first CSCC to DWFG was 20 (12–32) months. The estimated all-cause mortality rates in the cohort were 8.1%, 24.0%, and 31.4% at 1, 3, and 5 years, respectively. The most common cause of DWFG was malignancy-related (*n* = 13, 34.2%), of which 11 (84.6%) were directly attributed to CSCC. Estimated minimum CSCC-specific mortality after diagnosis was 1.5%, 7.6%, and 8.8% at 1, 3, and 5 years respectively. Other major causes of DWFG included infection (15.8%) and cardiovascular disease (15.8%).

Graft loss occurred in 17 patients (12.5%), with a median (IQR) interval of 34 (18–45) months after first CSCC. ISR was not associated with an increased risk of graft loss during follow-up (*P*: 0.23, Fisher test). The only episode of biopsy-proven rejection during follow-up occurred in a patient who had not undergone ISR.

*De novo* SOM was diagnosed in 10 patients (7.4%) during follow-up, with a median (IQR) interval from first CSCC of 16 (14–27) months. Of the cancers, 70% occurred within 2 years of the first CSCC, and half of those with new SOM had previously had multiple CSCC excised. Six of these patients died during follow-up. The estimated 1-, 3-, and 5-year survival for those diagnosed with SOM was 100%, 66.7%, and 50%, respectively. Of those patients with new SOM, 4 (40%) progressed to metastatic disease. One patient with history of breast cancer preceding the first CSCC also developed metastases at 14 months, and this was presumed to be breast cancer recurrence. The estimated 1-, 3-, and 5-year survival for those diagnosed with metastatic cancer (secondary to CSCC or SOM) during follow-up were 86.7%, 36.5%, and 26.1%, respectively.

Ninety-nine patients (72.8%) developed ≥1 composite poor outcome (graft loss, further CSCC, SOM, metastatic CSCC, or DWFG) during follow-up (Figure 2a), with most (66.7%) poor outcomes occurring within 2 years of the first CSCC (Figure 2b). Fifty-eight patients (42.9%) developed a poor outcome when further CSCC was removed from the composite (Figure 2f).

### Risk Factors for Further Lesion Development

**Figure 3:**
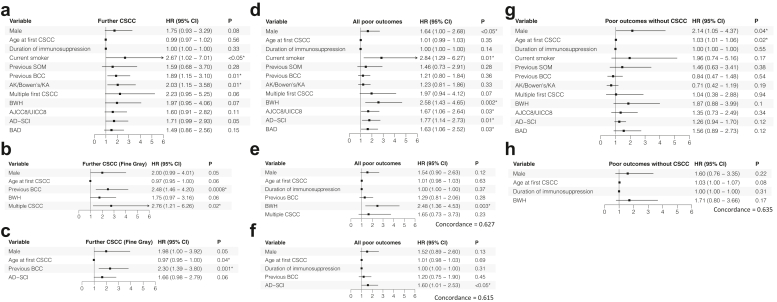
Univariate and multivariate modelling results. Forest plots showing results for risk of further CSCC from (a) univariate modelling and from multivariate modelling analysis with competing risk analysis using the Fine-Gray method, including either (b) BWH or (c) AD-SCI grading (multiple first CSCC is already part of AD-SCI and was not included as a separate variable). Forest plots show results for risk of any poor outcome from (d) univariate modelling and multivariate modelling analysis, including either (e) BWH or (f) AD-SCI. Forest plots show results for risk of any “poor outcome without CSCC” from (g) univariate modelling and (h) multivariate modelling analysis including BWH. Univariate modelling results are shown for all significant variables, as well as for important nonsignificant variables. Variables that were statistically significant or showed a trend towards significance (*P* < 0.10) on univariate analyses, as well as preselected clinically important risk factors (age and/or duration of immunosuppression) were included in the multivariate models, whereas smoking status and history of premalignant skin disease were excluded because of underreporting. Variables included the following baseline characteristics: male sex, age at first CSCC, duration of immunosuppression (in months), smoker (current vs. never), previous solid-organ malignancy, previous BCC, AK/Bowen’s/KA (history of premalignant skin lesions), multiple first CSCC, BWH (grades ≥ T2b), AJCC8/UICC8 (high-risk first CSCC, defined as grades T3 or greater), AD-SCI (grades 5 or 6), and BAD (“very high” risk). Significant results (P < 0.05) are indicated with an asterisk. AD-SCI, Actinic Damage and Skin Cancer Index; AJCC8/UICC8, 8th Edition of the American Joint Committee on Cancer/International Union Against Cancer staging system; AK, actinic keratosis; BAD, British Association of Dermatologists’ SCC guidelines; BWH, Brigham and Women’s Hospital classification; CI, confidence interval; CSCC, cutaneous squamous cell carcinoma; HR, hazard ratio; KA, keratoacanthoma; SOM, solid organ malignancy.

We evaluated characteristics present at time of first CSCC diagnosis associated with the development of further CSCC and poor outcomes, which might assist in risk stratification (Figure 3).

Smoking (HR: 2.7), previous BCC (HR: 1.9), and premalignant skin lesions (HR: 2.0) were predictive for developing further CSCC on Kaplan-Meier univariate analysis (Figure 3a). Male sex, high-risk CSCC, by either BWH (HR: 2.0) or the Actinic Damage and Skin Cancer Index (HR: 1.7), and multiple first CSCC (HR: 2.2) showed a trend toward increased risk, without reaching significance (*P*: 0.06–0.08).

Considering that smoking status was unknown for 53.7% of our cohort and documented history of premalignant lesions was likely underreported, both were excluded from further analysis. For multivariate modelling, we included statistically significant variables, or those trending toward significance (*P* < 0.10), as well as age and duration of immunosuppression, which are important clinical risk factors.^6^ History of BCC and multiple first CSCC remained independent predictors for further CSCC (Figure 3b and c).

Male sex (HR: 1.6), smoking (HR: 2.8) and high-risk CSCC (AJCC8/UICC8 HR: 1.7, BWH HR: 2.6, Actinic Damage and Skin Cancer Index HR: 1.8, British Association of Dermatologists’ CSCC guidelines HR: 1.6) were significant predictors for “all poor outcomes” (Figure 3d). Multiple first CSCC trended toward significance (HR: 2.0, *P*: 0.07). On multivariate analysis, high-risk CSCC (by BWH or the Actinic Damage and Skin Cancer Index) remained an independent predictor for “all poor outcomes” (Figure 3e and f). Because the most frequent poor outcome was further CSCC, analyses were repeated excluding this. Only male sex (HR: 2.1) and age at first CSCC (HR: 1.03) remained significant predictors of poor outcomes after excluding further CSCC, with a trend for high-risk CSCC by BWH (HR: 1.9, Figure 3g).

### International Validation of Outcomes

To externally validate our findings regarding significant morbidity after first posttransplant CSCC, we evaluated a single-center North American cohort of 79 SOTRs (including 46 KTRs) with a first CSCC during 2012 to 2020. Demographics and CSCC characteristics were similar between the cohorts (Supplementary Tables S5 and S6). The validation cohort had a lower cumulative duration of immunosuppression (64 vs. 136 months), but a greater proportion were receiving triple-immunotherapy (57.5% vs. 42.6%). Notably, index CSCC were more frequently low-risk (90.6% by BWH and 94.1% by AJCC). Follow-up duration was longer in the validation cohort (median: 59 months, IQR: 38–93). Comparably poor outcomes were observed after the first CSCC (Supplementary Table S7). The cumulative incidence of further CSCC at 1, 3, and 5 years were 29.4%, 58.3%, and 72.1%, respectively (Supplementary Figure S1A). These were not significantly different from our cohort (*P* > 0.05). Analysis of outcomes using Kaplan-Meier curves showed no significant differences between KTRs and other SOTRs (Supplementary Figure S1).

## Discussion

Malignancy is a leading cause of posttransplant morbidity and mortality, with CSCC specifically a major contributor to this. However, little is known about outcomes after first CSCC in KTRs in the modern era of immunosuppression. In addition, uncertainty regarding optimal management has been highlighted by the lack of expert consensus from a recent Delphi panel on managing low-risk CSCC in SOTRs or on immunosuppression modification after first CSCC.^25^

This retrospective study is the first to evaluate real-world management and outcomes after the first CSCC in a contemporary cohort of KTRs, and the largest ever study conducted over a single era of transplant practice. We report that this cohort, collected in a setting with previously underreported and increasing incidence of CSCC,^31^ develops a significant burden of morbidity after their index lesion, with up to half developing further CSCC and the majority developing ≥1 of the composite “all poor outcomes” within 5 years (many within 2 years). DWFG was observed in approximately a quarter of patients, with malignancy being the leading cause, surpassing both infection and cardiovascular mortality combined. Metastatic CSCC occurred in 13.3%, typically within 2 years of the first CSCC, with poor survival. Significant variation in early ISR and chemopreventative management was noted from transplant and dermatology practitioners, respectively. Although optimal secondary prevention strategies for this high-risk cohort remain unclear, we have identified common risk factors for both further CSCC and poor outcomes, namely male sex, multiple or high-grade CSCC, and history of BCC, which may identify patients most likely to benefit from targeted intervention.

Three similar studies were undertaken in North European SOTRs in the early 2000s, and provide a historical comparator.^7^^,^^8^^,^^30^ Contemporary KTRs are approximately a decade older at time of their first CSCC, reflecting the increasing age of patients at transplant.^11^ Male-predominance in the cohort was unchanged, reflecting sex-specific differences in chronic kidney disease prevalence alongside likely greater environmental CSCC risk factor exposure among men.^32^ The interval between transplant and first CSCC is broadly unchanged at approximately 11 years. In historic cohorts, most patients were receiving 3-agent immunosuppression, with three-quarters receiving azathioprine, whereas almost no KTR received antibody induction therapy.^7^^,^^8^^,^^30^ In contrast, induction therapy was used in most of our cohort. Observational studies suggest that T-cell depleting induction therapy received by a minority of our cohort, may specifically be associated with increased posttransplant skin cancer risk.^33^ Azathioprine, associated with enhanced CSCC risk in preclinical and observational studies,^34^ was only used by one-third of patients at the time of first CSCC, reflecting a shift toward mycophenolic acid–based antiproliferative therapy since the 2000s. Importantly, that outcome is unchanged from historical comparator studies despite azathioprine use reducing by two-thirds, as well as being the most commonly reduced immunosuppressive agent in this study, suggests that stopping azathioprine may not be as effective a secondary prevention strategy as it is a primary one.

Up to half of KTRs developed another CSCC within 3 years, representing a cumulative incidence almost identical to historic studies.^8^^,^^9^^,^^30^ Age is a major risk factor for CSCC development regardless of immunosuppression status, and the increasing age of KTRs may be hypothesized to increase CSCC burden. Conversely, newer immunosuppressive regimens are associated with lower carcinogenic risk.^12^^,^^13^^,^^16^ We conclude that, despite changes in KTR demographics and immunosuppressive regimens over the last 20 years, secondary outcomes following CSCC remain effectively unchanged.

One strength of the COAST study was the granular nature of patient-level data collected, permitting detailed evaluation of other relevant transplant outcomes. A composite poor outcome measure was used to capture the frequency of adverse events and highlight the significant morbidity and mortality among KTRs after first CSCC. These outcomes were identified as important and combined into a composite outcome because all are associated with a deleterious impact upon graft or patient survival. There is an increased risk of SOM in SOTRs after CSCC^10^ and 7.4% of patients developed an SOM during the study period, with a median interval of 16 months from first CSCC to diagnosis, with one-third dying within 3 years. For those who developed metastatic CSCC, the 3-year disease-specific survival was 24.5%. Although this is based on a small subset of patients and should be interpreted with caution, it is substantially lower than the 60% 3-year metastatic CSCC-specific survival reported previously,^35^ which may reflect the increased age and/or comorbidity burden of contemporary KTRs.^11^ Nearly one-quarter (23.3%) of our cohort died during the study period, with a median time to DWFG of 20 months. By comparison, a prospective cohort study of CSCC in the general population, which included immunosuppressed patients and SOTRs, reported a median overall survival of 51.8 months.^36^ Our study included graft loss as a key outcome, recognizing its significance to both patients and physicians.^37^ Fear of graft loss has been cited as a reason for hesitancy to reduce immunosuppression following CSCC; reassuringly, our data showed no increase in biopsy-proven rejection or graft loss among those who underwent ISR.

Our study demonstrates significant intra- and intercenter variation in immunosuppression modulation and chemoprevention use after a first CSCC. Centers appeared to have different strategies for secondary prevention, including ISR (range: 6%–50%), topical (range: 0%–58%) and systemic chemoprevention (range: 0%–25%). The use of topical therapies is likely underreported in clinical records, particularly among patients attending specialized dermatology clinics, where regular self-directed 5-fluorouracil treatment may not have been systematically documented. Our data provides the first real-world evidence for the lack of consensus around management after first CSCC, as indicated in a recent Delphi study.^25^

Difficulty stratifying KTRs at highest risk of poor outcomes after first CSCC may represent a significant cause of “therapeutic momentum” (defined as the reluctance to reduce therapy when continuation is not needed or not supported by evidence)^38^ regarding immunosuppression modulation for many clinicians. To address this, we identified readily available clinicopathological factors at the time of first CSCC, which are predictive of poor outcomes, including multiple index lesions, high-risk histopathology, and current smoking. Factors predictive of further CSCC likely relate in part to cumulative ultraviolet exposure, whereas high-risk histology may be a read-out of shared genetic and immunological host factors that predispose to further malignancy and/or mortality. These features may be clinically useful for identifying KTRs who may benefit most from prompt intervention.

The broad range of management strategies, as well as the varied clinical justifications given, reflect the ongoing lack of a consensus and limited evidence base in this area and likely contribute significantly to the apparent lack of improvement in outcomes observed in the contemporary era. Strikingly, only 1 patient was converted to an mTORi after diagnosis of first CSCC, despite trial evidence indicating greatest efficacy when this intervention is deployed at this stage.^4^^,^^39^ Low uptake of this approach likely reflects real-world hesitancy, resulting from the high burden of adverse effects with mTORi reported in clinical studies, leading to discontinuation in up to 80%.^20^

Although our study is limited by its retrospective nature, it is unlikely that further CSCC and other poor outcomes were missed. All KTRs undergo regular follow-up with their transplant and dermatology teams (the latter particularly after a diagnosis of CSCC) and receive education on skin self-surveillance and prompt reporting of skin lesions as part of routine care. The completeness of clinical records and histopathological results was further enhanced by the integrated nature of the health care system in the UK. The multicenter design of this study overcomes limitations from previous studies by identifying a cohort over a relatively short period, allowing the evaluation of management practices without confounding from era effects or local practice variations, often present in single-center studies. External validation was performed in an international cohort. Despite a higher prevalence of low-grade tumors and patients with additional transplant types (other than kidney) in the validation cohort, outcomes after the first CSCC remained equally poor, supporting the generalizability of our findings.

We conclude that KTRs with a first CSCC are at high risk for poor outcomes, leading to significant mortality and morbidity, which remain unchanged, if not worse, from historical studies. Continued clinical equipoise regarding secondary prevention in this population has led to a range of different strategies and the potential for undertreatment. These results add to the weight of evidence for more intensive dermatological and transplant follow-up, as well as earlier intervention to improve outcomes in this high-risk cohort. Furthermore, we identify factors that may permit a stratification approach to highlight KTRs who may benefit most from secondary prevention strategies after their first CSCC, while also urgently advocating for prospective studies to help guide clinicians managing this high-risk cohort.

## Disclosure

All the authors declared no competing interests.

## Supplementary Material

Supplementary File (PDF and CSV) **Figure S1.** Kaplan-Meir curves show event-free survival of international cohort (PDF). **Table S1.** Data Collection Proforma (CSV). **Table S2.** STROBE Checklist (PDF). **Table S3.** Patients ineligible for analysis (PDF). **Table S4.** Baseline characteristics of patients diagnosed with first cutaneous squamous cell carcinoma (CSCC), by contributing center (PDF). **Table S5.** Comparison of demographics with an international cohort (PDF). **Table S6.** Comparison of first CSCC characteristics with an international cohort (PDF). **Table S7.** Comparison of outcomes with an international cohort (PDF). **Table S8.** Clinical dataset (CSV).

## Footnotes

Supplementary File (PDF and CSV)
**Figure S1.** Kaplan-Meir curves show event-free survival of international cohort (PDF). **Table S1.** Data Collection Proforma (CSV). **Table S2.** STROBE Checklist (PDF). **Table S3.** Patients ineligible for analysis (PDF). **Table S4.** Baseline characteristics of patients diagnosed with first cutaneous squamous cell carcinoma (CSCC), by contributing center (PDF). **Table S5.** Comparison of demographics with an international cohort (PDF). **Table S6.** Comparison of first CSCC characteristics with an international cohort (PDF). **Table S7.** Comparison of outcomes with an international cohort (PDF). **Table S8.** Clinical dataset (CSV).
