# Supplementary material for: Clinical Equipoise and Unimproved Outcomes following Posttransplant Cutaneous Squamous Cell Carcinoma
Source: Kidney Int Rep. 2026 Apr 1;11(6):106522. doi: 10.1016/j.ekir.2026.106522 (PMC13187528; doi:10.1016/j.ekir.2026.106522)
Supplement: Supplementary File (PDF and CSV) — Figure S1. Kaplan-Meir curves show event-free survival of international cohort (PDF). Table S1. Data Collection Proforma (CSV). Table S2. STROBE Checklist (PDF). Table S3. Patients ineligible for analysis (PDF). Table S4. Baseline characteristics of patients diagnosed with first cutaneous squamous cell carcinoma (CSCC), by contributing center (PDF). Table S5. Comparison of demographics with an international cohort (PDF). Table S6. Comparison of first CSCC characteristics with an international cohort (PDF). Table S7. Comparison of outcomes with an international cohort (PDF). Table S8. Clinical dataset (CSV). [file mmc1.pdf]

Supplementary Material

Table of Contents

Supplementary Tables:

Supplementary Table S1. Data Collection Proforma (Excel)

Supplementary Table S2. STROBE Checklist (PDF)

Supplementary Table S3. Patients ineligible for analysis (PDF)

Supplementary Table S4. Baseline characteristics of patients diagnosed with first cutaneous squamous cell carcinoma (CSCC), by contributing centre (PDF)

Supplementary Table S5. Comparison of demographics with an international cohort (PDF)

Supplementary Table S6. Comparison of first CSCC characteristics with an international cohort (PDF)

Supplementary Table S7. Comparison of outcomes with an international cohort (PDF)

Supplementary Table S8. Clinical dataset (Excel)

Supplementary Figures

Supplementary Figure S1. Kaplan-Meir curves show event-free survival of international cohort (PDF)

Supplementary Tables

Supplementary Table S1. Data Collection Proforma

See separate file.

Supplementary Table S2. STROBE Checklist

	Item No	Recommendation	Page No
**Title and abstract**	1	(*a*) Indicate the study's design with a commonly used term in the title or the abstract	2-3
		(*b*) Provide in the abstract an informative and balanced summary of what was done and what was found	3-4
**Introduction**			
Background/ rationale	2	Explain the scientific background and rationale for the investigation being reported	5
Objectives	3	State specific objectives, including any prespecified hypotheses	6
**Methods**			
Study design	4	Present key elements of study design early in the paper	6-7
Setting	5	Describe the setting, locations, and relevant dates, including periods of recruitment, exposure, follow-up, and data collection	7
Participants	6	(*a*) Give the eligibility criteria, and the sources and methods of selection of participants. Describe methods of follow-up	7
		(*b*) For matched studies, give matching criteria and number of exposed and unexposed	NA
Variables	7	Clearly define all outcomes, exposures, predictors, potential confounders, and effect modifiers. Give diagnostic criteria, if applicable	7
Data sources/ measurement	8*	For each variable of interest, give sources of data and details of methods of assessment (measurement). Describe comparability of assessment methods if there is more than one group	7
Bias	9	Describe any efforts to address potential sources of bias	7-9
Study size	10	Explain how the study size was arrived at	7
Quantitative variables	11	Explain how quantitative variables were handled in the analyses. If applicable, describe which groupings were chosen and why	Supplementary table S2
Statistical methods	12	(*a*) Describe all statistical methods, including those used to control for confounding	8-9
		(*b*) Describe any methods used to examine subgroups and interactions	9
		(*c*) Explain how missing data were addressed	Supplementary table S4
		(*d*) If applicable, explain how loss to follow-up was addressed	7
		(*e*) Describe any sensitivity analyses	8

**Results**			
Participants	13*	(a) Report numbers of individuals at each stage of study—eg numbers potentially eligible, examined for eligibility, confirmed eligible, included in the study, completing follow-up, and analysed	10
		(b) Give reasons for non-participation at each stage	Supplementary table S4
		(c) Consider use of a flow diagram	NA
Descriptive data	14*	(a) Give characteristics of study participants (eg demographic, clinical, social) and information on exposures and potential confounders	10-12<br>Table 2
		(b) Indicate number of participants with missing data for each variable of interest	The total numbers of recorded data for each variable are stated in each table
		(c) Summarise follow-up time (eg, average and total amount)	16
Outcome data	15*	Report numbers of outcome events or summary measures over time	16-18<br>Table 4
Main results	16	(*a*) Give unadjusted estimates and, if applicable, confounder-adjusted estimates and their precision (eg, 95% confidence interval). Make clear which confounders were adjusted for and why they were included	16-19
		(*b*) Report category boundaries when continuous variables were categorized	NA
		(*c*) If relevant, consider translating estimates of relative risk into absolute risk for a meaningful time period	NA
Other analyses	17	Report other analyses done—eg analyses of subgroups and interactions, and sensitivity analyses	19-20
**Discussion**			
Key results	18	Summarise key results with reference to study objectives	20
Limitations	19	Discuss limitations of the study, taking into account sources of potential bias or imprecision. Discuss both direction and magnitude of any potential bias	24
Interpretation	20	Give a cautious overall interpretation of results considering objectives, limitations, multiplicity of analyses, results from similar studies, and other relevant evidence	21-23
Generalisability	21	Discuss the generalisability (external validity) of the study results	24
**Other information**			

Funding	22	Give the source of funding and the role of the funders for the present study and, if applicable, for the original study on which the present article is based	28

Supplementary Table S3. Patients ineligible for analysis

Reasons for exclusion	No. of patients
Care moved away from centre/abroad	3
First CSCC prior to study window	8
Non-cutaneous SCC	2
Patient receiving dialysis at time of CSCC	2
Incomplete data	2

CSCC, cutaneous squamous cell carcinoma; SCC, squamous cell carcinoma.

Supplementary Table S4. Baseline characteristics of patients diagnosed with first cutaneous squamous cell carcinoma (CSCC), by contributing centre

**Baseline Characteristics**	**All patients (n=136)**	**Centre A (n=32)**	**Centre B (n=12)**	**Centre C (n=19)**	**Centre D (n=8)**	**Centre E (n=17)**	**Centre F (n=23)**	**Centre G (n=4)**	**Centre H (n=21)**
Transplanting centre	NA	Yes	Yes	Yes	No	No	Yes	No	Yes
Age (years)	64 (57-72)	67 (55-71)	63 (58-68)	72 (58-74)	60 (52-68)	61 (57-70)	65 (52-73)	72 (69-73)	62 (60-73)
Sex									
Male	102 (75.0)	24	10	18	5	13	15	2	15
Female	34 (25.0)	8	2	1	3	4	8	2	6
Ethnicity									
White	128 (94.1)	32	12	15	8	17	20	4	20
Black	2 (1.5)	0	0	0	0	0	2	0	0
Asian	1 (0.7)	0	0	0	0	0	0	0	1
Not stated	5 (3.7)	0	0	4	0	0	1	0	0
Previous SOM [a]	11/135	6	1	1	0	1	0/22	1	1
Previous skin cancer [b]	46/135	14/31	3	4	2	6	11	3	3
Previous actinic keratoses [c]	67 (49.3)	17	3	5	6	13	13	3	7
Previous Bowen's/keratoacanthoma [d]	43/135	13	0	9	1	5	8	2	5
Smoking history									
Current	11 (8.1)	5	3	1	1	1	0	0	0
Ex-smoker	15 (11)	7	3	1	0	1	1	2	0
Never smoker	37 (27.2)	15	5	14	0	1	1	1	0
Not recorded/unknown	73 (53.7)	5	1	3	7	14	21	1	21
Total number of transplants									
One	107(78.7)	27	11	16	7	11	17	3	15
Two	23 (16.9)	4	1	2	0	4	6	1	5
Three	6 (4.4)	1	0	1	1	2	0	0	1
eGFR	41 (27-55)	33 (27-64)	40 (29-58)	44 (36-57)	37 (26-55)	42 (28-45)	32 (23-44)	38 (23-53)	44 (33-60)
Previous rejection[e]	21/134	0/31	6	2	3	1	6	0	3/20
Evidence of DSA prior to CSCC	11/134	0	1	2	1	3	4	0	0
Cumulative lifetime duration of IS (months)	136 (68-204)	166 (83-285)	83 (62-100)	149 (45-160)	138 (48-185)	120 (48-204)	162 (115-216)	83 (46-164)	156 (93-252)
Induction therapies[e]									
Basiliximab/equivalent	68/118	5/30	10	10/16	2/5	12	15/19	0	14/15
Alemtuzumab	21/118	10/30	0	1/16	3/5	5	0	2	0
ATG/Thymoglobulin	6/118	1/30	0	2/16	0	0	3/19	0	0
Other	4/118	0	1	3/16	0	0	0	0	0
None	19/118	14/30	1	0	0	0	1/19	2	1/15
Number of IS agents at diagnosis[e]									
One	8 (5.9)	3	0	5	0	0	0	0	0
Two	70 (51.5)	22	7	12	5	11	6	3	4
Three	58 (42.6)	7	5	2	3	6	17	1	17
Calcineurin/mTOR inhibitor									
Tacrolimus	104 (76.5)	21	11	17	6	15	13	3	18
Ciclosporin	18 (13.2)	6	0	0	1	1	8	0	2
Sirolimus	4 (2.9)	1	0	0	1	0	1	1	0
Nil	10 (7.4)	4	1	2	0	1	1	0	1
Antiproliferative[e]									
MMF/MPA	59 (43.4)	10	1	10	3	7	15	3	10
Azathioprine	46 (33.8)	17	5	2	4	1	5	1	11
Nil	31 (22.8)	5	6	7	1	9	3	0	0
Corticosteroid[e]	92 (67.6)	13	12	6	4	16	21	1	19
Year of diagnosis of first CSCC[e]									
2016	29 (21.3)	6	1	10	3	2	3	2	2
2017	20 (14.7)	8	1	1	1	0	6	0	3
2018	27 (19.9)	3	3	2	3	3	6	0	7
2019	36 (26.5)	9	6	3	0	6	6	1	5
2020	24 (17.6)	6	1	3	1	6	2	1	4

Number of CSCC removed at first episode[e]									
Single	127 (93.4)	31	11	14	8	16	22	4	21
Multiple	9 (6.6)	1	1	5	0	1	1	0	0
Early management									
IS reduction	39 (28.7)	9	3	3	4	1	8	1	10
Topical chemoprevention	28 (20.6)	0	7	5	0	6	9	0	1
Systemic chemoprevention	6 (4.4)	0	3	0	0	3	0	0	0

Values are expressed as number (percentage) or median (interquartile range), as appropriate.
Where data is missing, results have been expressed as a fraction.
ATG, anti-thymocyte globulin; CSCC, cutaneous squamous cell carcinoma; DSA, donor specific antibody; eGFR, estimated glomerular filtration rate; IS, immunosuppression; MMF, mycophenolate mofetil; MPA, mycophenolic acid; mTOR, mammalian target of rapamycin, SOM: solid organ malignancies.
[a]10/11 cancers were in remission at study baseline.
[b] Previous basal cell carcinoma (n=44) and/or melanoma(n=3) .
[c] Includes both clinical and histological diagnoses.
[d] Previous Bowen's disease (CSCC-in situ, histologically diagnosed in 37 patients and clinically diagnosed by a dermatologist in 4 patients) and/or keratoacanthoma (histologically diagnosed in 3 patients)
[e] Significant differences between centres (P <0.05), based on chi-squared test.

Supplementary Table S5. Comparison of demographics with an international cohort

	COAST	International
**Baseline Characteristics**	**All patients (n=136)**	**All patients (n=79)**
Age (years), median (IQR)	64 (57-72)	62 (54-70)
Sex		
Male	102 (75.0)	53 (67.1)
Female	34 (25.0)	26 (32.9)
Race		
White	128/131 (97.7)	78/78 (100)
Black	2/131 (1.5)	-
Asian	1/131 (0.7)	-
Previous skin cancer	46/135 (34.1)	31 (39.2)
Total number of transplants		
One	107 (78.7)	74 (93.7)
Two	23 (16.9)	5 (6.3)
Three	6 (4.4)	-
Types of Transplant		
Kidney	136 (100)	46 (57.5)
Lung	-	22 (27.5)
Heart	-	11 (13.7)
Liver	-	3 (3.8)
Pancreas	-	2 (2.5)
eGFR[a]	41 (27-55)	32 (21-56)
Cumulative lifetime duration of IS (months)	136 (68-204)	63.5 (31.7-162.0)
Number of IS agents at diagnosis		
One	8 (5.9)	1 (1.3)
Two	70 (51.5)	32 (40.0)
Three	58 (42.6)	46 (57.5)
Calcineurin/mTOR inhibitor		
Tacrolimus	104 (76.5)	62 (78.5)
Cyclosporin	18 (13.2)	8 (10.1)
Sirolimus	4 (2.9)	4 (5.1)
Everolimus	0	1(1.2)
Nil	10 (7.4)	4 (5.1)
Antiproliferative		
MMF/MPA	59 (43.4)	39 (48.4)
Azathioprine	46 (33.8)	26 (32.9)
Nil	31 (22.8)	14 (17.7)
Corticosteroid		
Prednisone	92 (67.6)	60 (75.9)
Number of CSCC removed at first episode		
Single	127 (93.4)	57/69 (82.6)
Multiple	9 (6.6)	12/69 (17.4)

Values are expressed as number (percentage), or median (interquartile range), as appropriate.

CSCC, cutaneous squamous cell carcinoma; DSA, donor specific antibody; eGFR, estimated glomerular filtration rate; IS, immunosuppression; MMF, mycophenolate mofetil; MPA, mycophenolic acid; mTOR, mammalian target of rapamycin.

[a] only collected for patients with kidney transplants

Supplementary Table S6. Comparison of first CSCC characteristics with an international cohort

	COAST	International
**Characteristics**	**All tumours (n=147)**	**All tumours (n=95)**
Location		
Head and neck	93 (63.3)	52/94 (55.3)
Trunk	12 (8.2)	11/94 (11.7)
Upper limb	37 (25.2)	19/94 (20.2)
Lower limb	5 (3.4)	12/94 (12.8)
Diameter (mm)	12 (9-20)	11 (6.0-15.0)
Depth (mm)	3 (1.9-5)	15 (8.0-30.0)
Invasion beyond the subcutis	8/128 (6.3)	8 (8.4)
Differentiation		
Well	40/145 (27.6)	72/85 (84.7)
Moderate	78/145 (53.8)	10/85 (11.8)
Poor	27/145 (18.6)	3/85 (3.5)
PNI	8/130 (6.2)	6 (6.3)
LVI	3/141 (2.1)	2 (2.1)
Clear margins after initial excision	117/146 (80.1)	84 (88.5)
BWH		
T1	77/133 (57.9)	67/85 (78.8)
T2a	41/133 (30.8)	10/85 (11.8)
T2b	15/133 (11.3)	6/85 (7.1)
T3	-	2/85 (2.4)
AJCC8/UICC8		
T1	91/134 (67.9)	68/85 (80.0)
T2	11/134 (8.2)	11/85 (12.9)
T3	32/134 (23.9)	6/85 (7.1)

Values are expressed as number (percentage) or median (interquartile range), as appropriate.
AD-SCI, actinic damage and skin cancer index; AJCC8/UICC8, the 8th edition of the American Joint Committee on Cancer/International Union Against Cancer staging systems; BWH, Brigham and Women's Hospital classification; LVI, lympho-vascular invasion; PNI, perineural invasion.

Supplementary Table S7. Comparison of outcomes with an international cohort

	COAST	International
**Outcome**	**All patients (n=136)**	**All patients (n=79)**
Follow up (months)	39 (26-52)	59 (38.0-93.0)
Poor outcomes [a]	99 (72.8)	40 (50.6)
Death	38 (23.3)	32 (40.5)
Time to death (months)	20 (12-32)	51.0 (35.3-65.0)
Causes of death		
CSCC-related	11 (28.9)	3 (9.4)
CVD	6 (15.8)	2 (6.2)
ARDS	-	5 (15.6)
Infection	6 (15.8)	1 (3.2)
Other malignancy	2 (5.3)	3 (9.3)
Other	2 (5.3)	-
Unknown	10 (26.3)	17 (53.1)
Graft loss	17 (12.5)	7 (8.8)
Time to graft loss (months)	34 (18-45)	28.0 (22.0-31.0)
Further CSCC	66 (48.5)	60 (75.9)
Median time to further CSCC (months)	13 (8-27)	18.0 (8.7-46.5)
Metastatic CSCC	18/135 (13.3)	10 (12.6)
Time to metastatic CSCC (months)	11 (7-12)	29.0 (6.5-62.5)
Solid-organ malignancy	10 (7.4)	4 (5.1)
Time to solid-organ malignancy (months)	16 (14-27)	53 (35.5-68.5)

Values are expressed as number (percentage) or median (interquartile range), as appropriate.
ARDS, Acute respiratory distress syndrome; CSCC, cutaneous squamous cell carcinoma; CVD, cardiovascular disease.
[a] Composite outcome including graft loss, further CSCC, solid-organ malignancy, metastatic CSCC, or death with a functioning graft.

Supplementary Figures

Supplementary Figure S1. Kaplan-Meir curves show event-free survival of international cohort

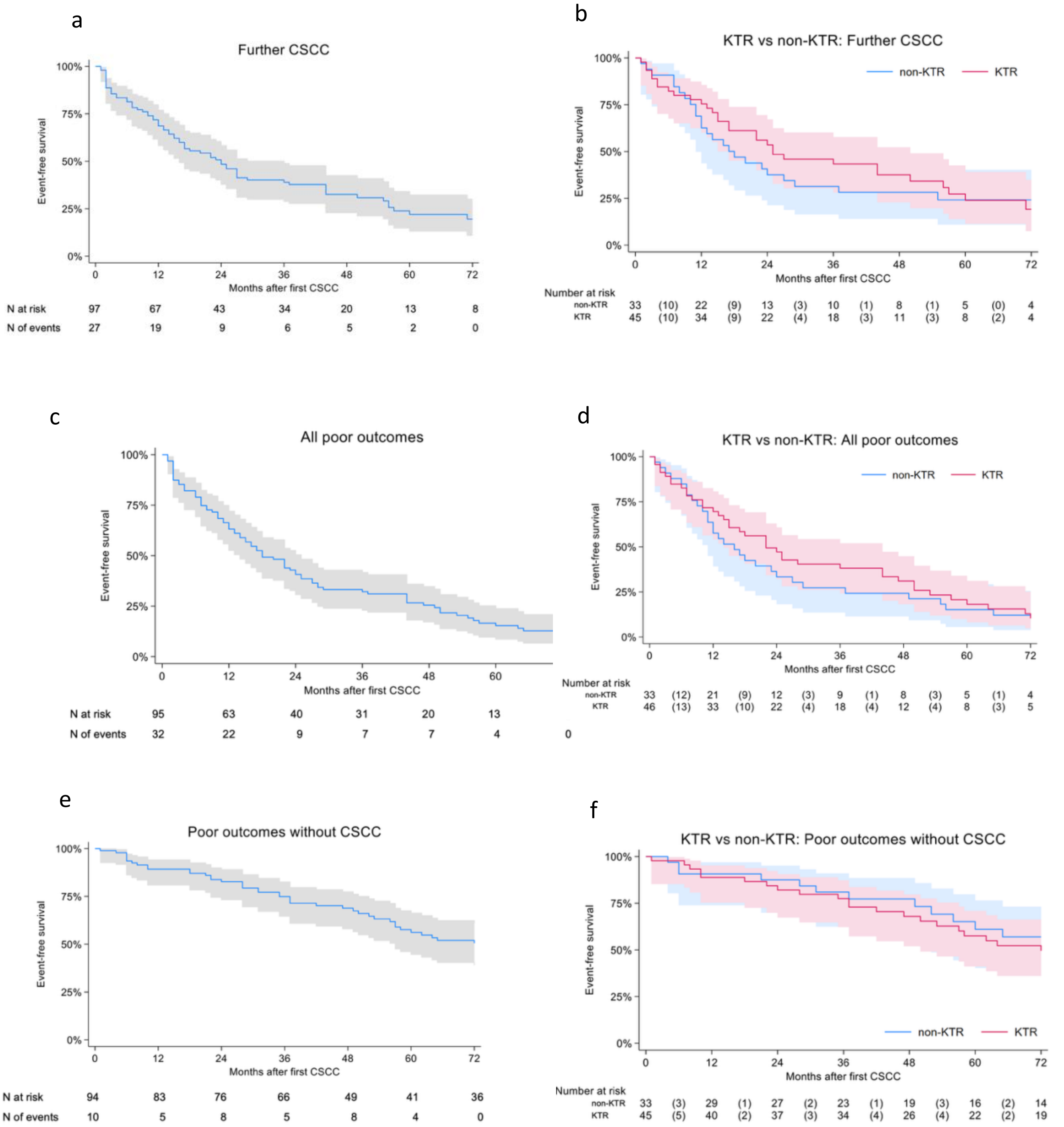

Kaplan-Meier curves show time to further CSCC for (a) the entire cohort and (b) stratified by transplant type: kidney transplant recipients (KTR) versus recipients of other organs (non-KTR). Kaplan-Meier curves show time to the composite 'all poor outcomes' for (c) the entire cohort and (d) stratified by transplant type. Kaplan-Meier curves show time to 'poor outcomes without CSCC' for (e) the entire cohort and (f) stratified by transplant type. CSCC: cutaneous squamous cell carcinoma; KTR: kidney transplant recipient.
